# Efficacy and safety of suanzaoren decoction for chronic insomnia disorder in adults: study protocol for randomised, double-blind, double-dummy, placebo-controlled trial

**DOI:** 10.1136/bmjopen-2016-014280

**Published:** 2017-04-04

**Authors:** Qi-Hui Zhou, Hui-Lin Wang, Xiao-Li Zhou, Meng-Bei Xu, Hong-feng Zhang, Li-bo Huang, Guo-qing Zheng, Yan Lin

**Affiliations:** Department of Neurology, The Second Affiliated Hospital and Yuying Children's Hospital of Wenzhou Medical University, Wenzhou, China

**Keywords:** Suanzaoren decoction, chronic insomnia disorder, study protocol

## Abstract

**Background:**

Insomnia disorder is defined as a combination of dissatisfaction with sleep quantity or quality and a significant negative impact on daytime functioning. Chronic insomnia disorder refers to clinical symptoms of persistent insomnia at least three nights a week for at least 3 months. Prevalence estimates of insomnia disorder range from 12% to 20% in the adult population, with approximately 50% having a chronic course. The potential side effects of hypnotic medications hinder their clinical application. Thus, traditional Chinese medicine is considered as an alternative option for treating insomnia.

**Objective:**

To evaluate the efficacy and safety of suanzaoren decoction (SZRD), a classic Chinese herbal prescription, for adult chronic insomnia disorder.

**Methods/analysis:**

This is a randomised, double-blind, double-dummy, placebo-controlled clinical trial. A total of 150 patients with chronic insomnia disorder are randomised, allocated in a ratio of 1:1:1 to three groups: intervention group, control group and placebo group. The intervention group receives SZRD granule plus zolpidem tartrate (ZT) placebo; the control group receives ZT tablet plus SZRD granule placebo; and the placebo group receives ZT placebo and SZRD granule placebo. The patients receive medicine or placebo for 5 weeks and are followed up at 20 weeks. The primary outcome measures are polysomnography and Pittsburgh Sleep Quality Index. Secondary outcome measures are the Insomnia Severity Index, sleep diary and safety assessment. Outcomes will be assessed at baseline and after treatment.

**Trial registration number:**

ChiCTR-IOR-16009198. pre-results.

**Strengths and limitations of this study**

- This is a well-designed study to assess the efficacy and safety of suanzaoren decoction for chronic insomnia disorder in adults.
- The results from this randomised, double-blind, double-dummy, placebo-controlled clinical trial will provide new evidence of the efficacy of suanzaoren decoction for insomnia.
- This study will provide a herbal prescription for adult chronic insomnia disorder after cognitive and behavioural therapy for insomnia based on the American College of Physicians’ recommended guideline.
- One limitation is the participant self-rating scales, which might exaggerate the severity of the sleep disorder.
- Another limitation is that the trial is implemented in only one hospital in Chinese subjects; this may limit its generalisability.

## Introduction

Insomnia disorder is defined as a combination of dissatisfaction with sleep quantity or quality and a significant negative impact on daytime functioning. Based on the International Classification of Sleep Disorder (ICSD)-3 of the American Academy of Sleep Medicine, insomnia is present when one or more of the following persistent symptoms are seen: difficulty initiating sleep, difficulty maintaining sleep and early-morning awakening with inability to return to sleep.^1^ Chronic insomnia disorder refers to these symptoms that cause clinically significant functional distress or impairment on at least three nights a week for at least 3 months, excluding other sleep, medical or mental disorders.^2^

Insomnia is highly prevalent in the general population and commonly encountered in medical practices. Prevalence estimates of insomnia disorder range from 12% to 20% in the adult population, with approximately 50% having a chronic course.^3^ The latest guideline from the American College of Physicians (ACP) recommends that cognitive and behavioural therapy for insomnia (CBT-I) should be the initial treatment for chronic insomnia disorder in all patients.^2^ If CBT-I alone is unsuccessful, the ACP advises that clinicians discuss with patients whether to combine it with pharmacological therapy, including benzodiazepines and non-benzodiazepine hypnotic agents.^2^ However, the hypnotic agents have a range of undesirable side effects, such as memory and performance impairment, residual sedation, undesired behaviours during sleep, falls, somatic symptoms and drug interactions.^4^ ^5^ Thus, an increasing number of patients with insomnia resort to various kinds of complementary and/or alternative medicine (CAM) worldwide, including acupuncture, meditation, massage and Chinese herbal medicine.^6–9^

Traditional Chinese medicine, a form of CAM, has been widely used to treat insomnia in China for a 1000 years.^10^ Nowadays, it is still commonly being used for the patients with insomnia disorder in China and elsewhere around the world.^11^ ^12^ Suanzaoren decoction (SZRD) also has a long history of use as a classic herbal prescription for combating insomnia and was first documented in the classical Chinese text *Jingui Yaolue* (*Synopsis of Prescriptions of the Golden Chamber*) by Zhong-Jing Zhang (AD 152–219).^13^ It is composed of five kinds of Chinese herbal medicines (table 1): Suanzaoren (*Semen ziziphi spinosae,* seed of wild jujube), Fuling (*Poria,* Hoelen), Chuanxiong (*Ligusticum,* Chuanxiong Rhizoma), Zhimu (*Anemarrhena aspodeloidea,* Anemarrhena) and Gancao (*Glycyrrhiza glabra L.,* Radix Glycyrrhizae), all of which are recorded in the Chinese Pharmacopoeia (V.2015).

**Table 1:** Overview of Suanzaoren decoction

Chinese name	Common name	Latin name	Species/family	Amount (%)
Suanzaoren	Spine date seed	*Semen ziziphi spinosae*	The dried ripe seeds of Ziziphus jujuba Mill. var. spinosa (Bunge) Hu ex H. F. Chou/Rhamnaceae	20 g (41%)
Gancao	Liquorice root	*Glycyrrhiza glabra L.*	The dried roots or rhizome of G uralensis Fisch. or G inflata Bat. or G glabra L./Leguminosae	3 g (6%)
Zhimu	Common anemarrhena rhizome	*Anemarrhena aspodeloidea*	The dried rhizome of Anemarrhena asphodeloides Bge./Liliaceae	10 g (20%)
Fuling	Indian buead	*Poria*	The dried sclerotia of P cocos (Schw.) Wolf/Polyporaceae	10 g (20%)
Chuanxiong	Chuanxiong rhizoma	*Rhizoma Ligustici Chuanxiong*	The dried rhizome of L chuanxiong Hort./Umbelliferae	6 g (13%)

Experimental studies have found that SZRD increases spontaneous sleep activity and its mechanisms are considered to be mediated through the serotonergic system.^14–16^ Spinosin and jujubosides are the main active compounds of Semen ziziphi spinosae contributing its sedative and hypnotic effects on insomnia.^17–19^ The National Health Insurance programme in Taiwan found that SZRD is the most frequently prescribed Chinese herbal formula for treating insomnia.^20^ A meta-analysis in our group has demonstrated that the current evidence is insufficient to support the efficacy and safety of SZRD for insomnia due to lack of high-quality randomised controlled trials.^21^ In 2015, a randomised, double-blind, placebo-controlled trial was conducted by Chan *et al*,^22^ indicating that SZRD is effective for improving sleep quality and quantity in methadone-maintained patients with sleep disturbances. However, that study specifically focused on methadone-maintained sleep disturbances. Therefore, the objective of our study is to evaluate effectiveness and safety of SZRD for adult chronic insomnia by a rigorous randomised controlled trial.

## Method

### Trial design

**Figure 1:**
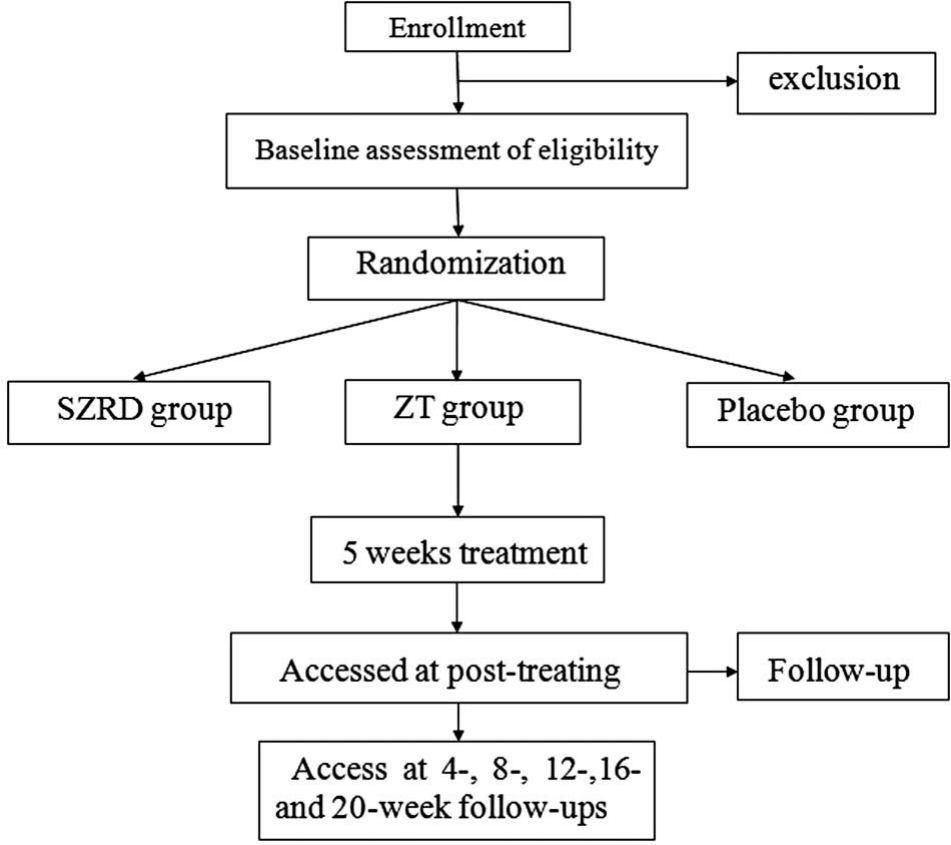
Flow diagram. SZRD, Suanzaoren decoction; ZT, zolpidem tartrate.

This is a double-blind, double dummy, placebo-controlled trial. The study will be conducted in the Second Affiliated Hospital of Wenzhou Medical University, a teaching hospital in China. Patients with sleep dissatisfaction undergo a standardised baseline evaluation before treatment comprising detailed history taking, physical examination and laboratory testing. The scale will be assessed by a professional psychological evaluator. The hospital clinical laboratory and the sleep monitoring room are responsible for biochemical and sleep quality indicator detection, respectively. All included patients are randomly divided into three groups; every patient will receive CBT-I as an initial therapy. Additionally, three groups are designed to receive SZRD granule plus zolpidem tartrate (ZT) placebo, ZT tablet plus SZRD granule placebo or both of the two placebo treatments, respectively. The efficacy and safety of the SZRD granule is assessed after 5-weeks’ treatment and at 20-weeks’ follow-up after drug withdrawal. The trial is conducted in accordance with the World Medical Association Declaration of Helsinki and China's regulations and guidelines on good clinical practice. Ethical clearance for the trial was obtained from the ethics committee of the Second Affiliated Hospital of Wenzhou Medical University. After a full explanation by the clinicians, written informed consent will be obtained from the included subjects before intervention. The trial was registered at the Chinese Clinical Trial Registry (ChiCTR-IOR-16009198) on 13 September 2016 and will be carried out from January 2017 to December 2017. The study design is shown in figure 1.

### Sample size

The trial aims to detect if SZRD is as effective as ZT for treating sleep disorders. We deem a clinically significant difference to be an average two-point reduction on the Pittsburgh Sleep Quality Index (PSQI) scores in the intervention group in comparison with the control group. According to a previous study by Shi *et al*,^23^ the global score of PSQI after 12 weeks' treatment is 4.52±3.218 in the intervention group and 10.42±3.214 in the control group, respectively. On the basis of a 0.9 power to detect a significant difference (α=0.01, two-sided), 44 participants will be required for the two groups and in a 1:1:1 ratio, we enrolled 44 patients in the placebo group. Allowing for a 10% withdrawal rate, we plan to include 150 patients in the whole trial.

### Participants and recruitment

#### Inclusion criteria

Subjects are included in the study if they meet the following criteria: (i) >18 and <80 years old; (ii) chronic insomnia disorder diagnosed by two clinical attending doctors according to the ICSD-3 criteria;^1^ the symptoms must be present for at least three nights a week for at least 3 months; (iii) after shared decision-making, clinicians decide to add pharmacological therapy if the CBT-I^24^ alone is unsuccessful; (iv) have good compliance and are willing to accept the test; (v) no mental disease and not using psychoactive medications; (vi) sign the written informed consent form for the clinical trial.

#### Exclusion criteria

Individuals are excluded if one of the following criteria is met: (i) diagnoses of severe hepatic, renal or thyroid dysfunction or cardiovascular diseases; (ii) have a severe psychiatric disorder or a history of major psychiatric disorder (eg, depression, anxiety, autism, schizophrenia and so on); (iii) diagnoses of other sleep disorders such as narcolepsy, obstructive sleep apnoea syndrome or restless legs syndrome; (iv) taking sleep medications, psychotherapy or acupuncture for insomnia within a month; (v) taking part in another clinical trial; (vi) diagnosed with severe, life-threatening chronic sleep disorders; (vii) not suitable for the study based on a physician's evaluation; (viii) pregnant, breast-feeding or juvenile.

#### Exit criteria

Patients will leave the trial when one of the following criteria is met: (i) are poorly compliant or pregnant; (ii) insomnia is aggravated during the trial, resulting in serious problems such as severe depression, mania and suicidal tendency, which need emergency interventions; (iii) appearance of other problems which need to be dealt with. Participants may withdraw from the study at any time for any reason.

### Concomitant treatments and forbidden drugs

Treatment must not be combined with other sleep medications in the study. Acupuncture therapy, psychotherapy and any drugs and diet such as psychopharmacological drugs, opioids, anxiolytic agents, coffee and alcohol, which may induce sedative or hypnotic effects, are also not allowed in this trial. Patients who have already taken medicine that does not act upon the central nervous system before the trial can be included. Nevertheless, all combined administration should be recorded on the case report form.

### Randomisation and allocation concealment

The randomisation will be performed by an independent statistician. The randomisation sequence is generated by the use of SAS software and randomisation numbers are kept in a predetermined computer-made opaque sealed envelope. The random numbers are divided into three groups sequentially; the group numbers are printed inside the envelopes. The sealed opaque envelopes are properly preserved by the designer and the statistician until the end of the trial. All envelopes will be numbered consecutively and connected. Clinicians who screen the eligible patients will open the envelopes according to the patients' screening sequence numbers and then assign the patients to group A, B or C in accordance with the group number inside.

### Blinding

This is a double-blind (with patients and clinicians blinded) trial. The evaluation of participants and analysis of results will be performed by professionals. Treatment assignments will not be revealed until the whole process is complete. If patients have severe adverse events, clinicians will unblind the patients as an emergency and provide relevant treatment. To achieve blinding, all three groups will use the same kind of packaging to encase the drug or placebo. Size, colour, shape, taste, smell and packaging of the placebo are made identical to that of the corresponding medicine by adding artificial pigment.

### Intervention

All researchers are clinical doctors with certification in neurology for at least 3 years and receive standardised training for the diagnostic interview before the start of the trial. Two clinicians make the diagnosis of chronic insomnia disorder. Participants in the intervention group will receive SZRD granule plus ZT placebo orally twice a day for 5 weeks. Participants in the control group will take ZT tablet plus SZRD granule placebo twice a day for 5 weeks. Participants in the placebo group are about to receive ZT placebo and SZRD granule placebo twice a day for 5 weeks. Patient visits are required as often as once a week. The SZRD granule placebo is composed of 98% maltodextrin, 2% caramel and very little bitterant. ZT placebo is made of starch. SZRD granule and its placebo are produced by China Resources Sanjiu Medical & Pharmaceutical Co, Ltd and can be preserved for 2 years. ZT tablet is provided by Senofi (Hangzhou) Pharmaceutical Co, Ltd at a dosage of 10 mg. The SZRD granule placebo and ZT placebo are similar to the SZRD granule and ZT in size, colour, shape, taste, smell and packaging, respectively.

### Follow-up

All included patients will be re-evaluated at 4-, 8-, 12-, 16- and 20-week follow-ups through phone calls or as an outpatient. Patients whose sleep quality worsens will receive a supply of relevant medicine and a written withdrawal schedule.

### Outcome measures

#### Primary outcomes

##### Polysomnography

Polysomnography (PSG) is the ‘gold standard’ for sleep assessment and is the only instrument that objectively evaluates sleep using quantifiable data, such as total sleep time, sleep onset latency, waking after sleep onset, rapid eye movement and non-rapid eye movement sleep.^25^ All included patients will be assessed for two nights (one night at baseline and one night after the treatment phase) in a sound-attenuated, light-controlled sleep laboratory. During the assessment, patients are allowed to sleep voluntarily based upon their habitual sleep time. Sleep will be recorded with standard equipment from 22:00 to 7:00 each time. All PSG data will be further collected and processed using an E-series digital system (Compumedics, Abbotsford, Australia) by an experienced PSG technologist blinded to the treatment assignment.

##### Pittsburgh Sleep Quality Index

PSQI is a self-report questionnaire measuring sleep quality and disturbance over a period of 1 month.^26^ The items are divided into seven ‘component’ scores, and a lower global score reflects a better quality of sleep. As sleeping medication is used in the intervention and control groups but not in the placebo group, we delete the item, ‘use of sleeping medication’, for comparability of the three groups. The six remaining items used to rate sleep are sleep quality, sleep latency, sleep duration, habitual sleep efficiency, sleep disturbances and daytime dysfunction.

#### Secondary outcomes

##### The Insomnia Severity Index

The Insomnia Severity Index is a seven-item self-rated instrument that is used to assess the nature, severity and impact of insomnia during the previous 1 month.^27^ ^28^ The seven items are sleep onset, sleep maintenance, early morning awakenings and satisfaction with current sleep pattern, interference of sleep difficulties with daytime functioning, ability to notice sleep problems and distress caused by sleep disorder. The total score ranges from 0 to 28, with higher scores indicating more severe levels of insomnia. Each item is rated on 0–4 scale (0=no problem, 4=very severe problem). Insomnia severity is classified as: no clinically significant insomnia (total score: 0–7), subthreshold insomnia (total score: 8–14), moderate clinical insomnia (total score: 15–21) and severe clinical insomnia (total score: 22–28).

##### Sleep diary

Estimates of awake and sleep times are obtained daily using a sleep diary. Participants report information about their night’s sleep, including bedtime, wake time, sleep-onset latency, total sleep time, number and length of nocturnal awakenings, subjective rating of sleep quality and other factors that may influence sleep. Participants with missing data are contacted by phone to remind them to fill out the diary daily. Sleep diaries are obtained for 1 week at baseline, at each subsequent assessment period and for the entire treatment period.

### Safety assessments

Safety will be assessed by renal function test, liver function test, routine blood test, routine urine test, routine stool test and electrocardiogram. These indicators are detected during the period of screening and after 5-weeks’ treatment. Adverse events are defined as all deaths, suicide attempts, unexpected signs or symptoms and any physical changes. The occurrence of any adverse events in trial participants will be recorded in the case report forms during each patient visit. We will withdraw patients who have severe adverse events, as it is unsafe for them to continue the trial. Meanwhile, we will give them relevant medical care and follow them up until the reaction has terminated.

### Data analyses

Data analysis will be performed by professional statisticians using SPSS software. An intention-to-treat analysis will be conducted for patients who have received treatment at least once. We will perform a sensitivity analysis using various imputation methods to detect if the results are robust for different assumptions about the missing data. The per-protocol analysis will be restricted to patients who do not violate the protocol and complete the programme. We use mean±SD for continuous variables and percentages for categorical variables. Analysis of variance is performed on categorical variables and Pearson's χ^2^ test on continuous variables. The study will set an α level of 0.05 two sided for all statistical tests. 95% CI will be used for continuous variables.

### Ethics and dissemination

The protocol has been approved by the ethics committee of the Second Affiliated Hospital of Wenzhou Medical University (No. 201656) and has been registered with the Chinese Clinical Trial Registry (ChiCTR-IOR-16009198). The trial will help to demonstrate if SZRD is effective and safe for patients with chronic insomnia disorder. We will publish the results of this study in peer-reviewed journals to ensure widespread dissemination.

## Discussion

To our knowledge, this is the first study protocol of a randomised, double-blind, double-dummy, placebo-controlled trial testing SZRD for chronic insomnia disorder in adult. This trial aims to evaluate the efficacy and safety of SZRD in comparison with ZT or placebo. To facilitate high validity and reliability, a strict quality control and high-quality methodology is indispensable. We describe in detail the method of allocation concealment, recruitment, randomisation and data collection. Additionally, objective sleep measurement, such as PSG, is used to assess insomnia remission. Currently, the ACP recommends that patients with insomnia should receive CBT-I as first-line therapy. Thus, we provide every patient with CBT-I at the start and include only those subjects who are insensitive to CBT-I alone. The results from this trial may provide evidence on the effectiveness and safety of SZRD.

Our study has some limitations and strengths. One weakness is the participant self-rating scales, which might exaggerate the severity of sleep disorder. Another limitation is that the trial is implemented in only one hospital. Notwithstanding these limitations, the results from this study will provide new evidence about SZRD from a well-designed trial. In addition, this study will provide a herbal prescription for adult chronic insomnia disorder after CBT-I based on the ACP's recommended guideline.
